# Special Correspondence of the British Medical Journal—Paris, Vienna, Berlin

**Published:** 1888-06

**Authors:** 


### ABSTRACTS AND EXTRACTS.

### SPECIAL CORRESPONDENCE OF THE BRITISH MEDICAL JOURNAL.

#### PARIS.

*Disinfection of Tuberculous Sputa—Transmission of Tuberculosis from the Human Subject to the Dog—Experiments on Hereditary Alcoholism—Chloral in Diphtheria.*—At the meeting of the Société de Médecine Publique et d'Hygiène Professionnelle, February 22, 1888, M. Grancher gave the results of researches made by himself and Dr. De Gennes, on the disinfection of tuberculous sputa. The air exhaled by tuberculous patients does not affect animals, therefore it does not contain bacilli. M. Grancher and Dr. De Gennes disinfected tuberculous sputa with carbolic acid at 5 per cent.; potassium, at 5 per cent.; sulphate of copper, at 5 per cent.; chloride of zinc, at 5 per cent.; corrosive sublimate, at 1 per cent. Guinea-pigs were submitted to injections of sputa thus disinfected. The results were not encouraging; the corrosive sublimate alone appeared to kill the tuberculous bacillus. It is difficult, however, and even dangerous, to use it at 1 per cent. Guinea-pigs were inoculated with sputa which had been mixed with hot water, and exposed to a temperature of 60°, 80°, and 100° C. It was discovered from these experiments that the bacilli resist water at 60°, are nearly always killed at 80°, and at 90° and 100° are always killed. MM. Geneste and Herscher have constructed an apparatus to be placed near each hospital ward. M. Lailler asked if it would not be possible to have one apparatus only for each hospital, on account of the expense; and if the apparatus could not be heated by steam instead of gas. Dr. Ollivier remarked that M. Grancher's experiments proved the insufficiency of the ordinary means of disinfection, such as chloride of zinc. Meat should be always well cooked, and when prescribing it raw, the physician should ascertain that only non-tuberculous mutton was used. M. Grancher replied that raw meat was not dangerous. The experiments of M. Nocard prove that the bacillus of tuberculosis rarely exists in the flesh or juices of animals that die of this disease unless there be tuberculous glands.

Dr. Bourgougnon publishes in the *Journal de Médecine*, March 18, 1888, a case in which tuberculosis was transmitted from a human being to a dog. "The patient had suffered for some time from a slight, dry, exhausting cough, feverishness at night, and intermittent pains in the thorax. His father died of an affection of the heart, his mother of phthisis; two sisters died from tuberculosis, one at 19, the other at 27 years of age. The patient, when first examined, presented no symptoms of tuberculosis. After some months, however, cough and fever increased; the patient became breathless after going up stairs, lost flesh, and had nocturnal sweats. In the month of February, 1886, distinct crepitations and dullness were heard under the left clavicle. Finally, in October, 1886, he died with all the symptoms of pulmonary tuberculosis, the disease having lasted about fifteen months. On first visiting the patient I noticed a dog, whose extreme thinness attracted my attention. This dog barked at me whenever I entered the room, and I observed that after barking he was seized with a fit of coughing. The persistence of this symptom, together with the continual loss of flesh, awakened my curiosity, and reminded me of the cases of contagion reported by Dr. Léon Petit. I advised the patient to have the dog sent to a veterinary surgeon. This was done, with the result that the animal was pronounced to be tuberculous. The dog became worse and worse, extremely thin, its hair rough, and as stiff as if it had been brushed backwards. The owner would have had it killed, as its cough was distressing, the expectorations frequent, and the appetite quite lost, but I asked him to let the disease take its course, that the veterinary surgeon and I might make a *post-mortem* examination. Meanwhile I learned that the dog had been given to the patient's sister as a puppy, and during her illness had been literally brought up in her bed. After her death my patient had taken care of the dog, which had thus been brought up in contact with two tuberculous patients. The dog died in the last stage of phthisis in November, 1886. M. Plouchart, veterinary surgeon, of Tours, made the *post-mortem* examination in my presence. The lungs were riddled with tubercles; the left lung contained many patches of softening. The peritoneum was covered with miliary tubercles. The left kidney presented a great number of miliary tubercles; in the right we found four or five tuberculous nodules in process of softening. The stomach and intestines were healthy. It is evident that the disease was transmitted to the animal by my patient and his stster, tuberculosis being of very rare occurrence in dogs. The mode of transmission is not quite clear. The dog doubtless absorbed bacilli from swallowing the sputa or vomit of the patients, yet the healthy condition of the stomach and intestines would point to its having acquired the disease by the respiratory organs."

At the meeting of the Académie des Sciences on March 5, MM. A. Mairet and Combemale related their observations on the degenerative hereditary influence of alchohol. Twelve puppies were born of a dog submitted to chronic intoxication with alcohol, and a young, healthy bitch with no defect. Two were stillborn, three died accidentally; the seven others succumbed to various diseases—epileptic attacks, verminous enteritis, pulmonary and peritoneal tuberculosis. At the necropsy, lesions, evidently arising from alcoholic degeneration, were found—thickening of the bones of the skull, premature closing of fontanelles, adhesion of the dura mater to the cranial bones, difference in weight of the cerebral hemispheres, fatty degeneration of the liver. A strong, intelligent bitch, during the last three weeks of gestation, was intoxicated with absinthe, and gave birth to six puppies, of which three were stillborn; two were well developed physically, but were not at all intelligent; the third—a bitch X—grew slowly, presenting defective intelligence and a remarkable degree of anosmia. X, presenting symptoms of degeneration more especially of the nervous system, was coupled with a strong, intelligent dog. She gave birth to three puppies, one of which had flat foot, atrophy of several of the toes, wolf's mouth; a second died of marasmus; the third was attacked with atrophy of the hind legs. From the latter case it would appear that the degenerative influence of alcohol is greater in the second than in the first generation.

Dr. Adolphe Mercier (of Besançon) has utilized the antiseptic properties of chloral in the treatment of diphtheria in children with excellent results. He proceeds in the following manner: when the tongue is thick, coated, and swollen, the child is given an emetic of ipecacuanha, without tartrate of antimony, which causes prostration. When vomiting has ceased, sirup of chloral, prepared according to the French codex (at 5 per cent.), is given every half-hour, in doses of two, three, or five grammes, according to the age of the patient. It is better to begin by giving the child rather large doses in order to keep him in a state of somnolence, as medicine is then more easily administered. The submaxillary and anterior regions of the neck are covered with a thick layer of belladonna ointment, and wrapped up in cotton-wool. In order to keep the throat impregnated, and prevent the chloral from causing pains in the stomach, the patient is given something to drink every time before, and not after, taking that remedy. As long as he continues taking chloral, to which M. Mercier regularly adds sirup of bark, the patient may eat and drink whatever he fancies—milk, wine, lemonade, or any solid food. This treatment must be rigorously carried out during forty-eight hours. No change ever takes place before twenty-four hours. In forty hours the false membranes begin to come away, and have completely disappeared by the forty-eighth hour. In the case of patients with fair hair and white skin, they may only come away on the third day. After the separation of the false membranes, the sirup of chloral causes smarting in the throat, and must then be stopped. In rare cases the loosening of the false membranes leaves the tonsils red or swollen. An astringent gargle will do away with any swelling of that kind. A thick coating of vaseline applied to the neck will get rid of any pustular eruption caused by the ointment. If during the illness any spasms or dyspnœa occur, the throat is painted with a 1 in 50 solution of hydro-chlorate of cocaine. Prescribed in the last stages of the disease, when the voice is gone, and laryngeal diphtheria has set in, the chloral treatment would be rather injurious than otherwise.

### VIENNA.

*Excision of Indurated Tissue and Enlarged Glands in Syphilis—Erythrophlœin.*—Dr. Neumann showed a patient from whom he had a long time ago removed the primary hard sore, and the enlarged lymphatic glands, on the thirty-first day after syphilis had been contracted. A macular rash and desquamating papules nevertheless appeared over the lower part of the abdomen on the fifty-third day. The patient, who seemed to be quite cured after a long course of treatment, came again under the care of Professor Neumann, on April 16 of the present year, with the following syphilitic lesions: gummatous orchitis; syphilitic ulcers and gummata on the posterior wall of the pharynx and the soft palate, as well as periostitis of the tibia. This observation sufficiently proved, in the opinion of the speaker, that extirpation of the primary indurated chancre and the lymphatic glands not only did not prevent the spread of the syphilitic affection, but that it did not even make the course of the disease milder.

Dr. Adolf Onodi gives, in the *Orvosi Hetilap*, a summary of his observations on twelve patients, and concludes that erythrophlæin is undoubtedly a powerful anæsthetic, the effect of which comes on more slowly, but, on the other hand, lasts much longer, than that of cocaine. Local anæsthesia persists for eight hours and even longer from the time of instillation. It has, nevertheless, very disagreeable after-effects, such as headache, giddiness, sometimes even fainting fits; in particular it dims the cornea for several hours. When applied to the nose, the mouth, the soft palate, and the external orifice of the urethra, a 0.20 per cent. solution caused a slight sensation of burning. He did not venture to use stronger solutions.

BERLIN.

*Dangers of Antiseptics.*—At the last meeting of the Berliner Medicinische Gesellschaft, Dr. Emil Senger read a paper on the influence of antiseptic remedies on the organs of the body, with special reference to operations on the kidney. It is well known that after nephrectomy, or even nephrotomy, many patients die with symptoms of uræmia or anuria, even when it had been ascertained beforehand by careful examination that the other kidney was quite healthy and capable of secreting the necessary amount of urea. Dr. James Israël, chief surgeon of the Berlin Jewish Hospital, has propounded a very complicated theory as to certain nervous sympathies between the two kidneys, whereby an operation on one may give rise to degeneration of the other. Senger has now proved by experiments on rabbits and dogs that our antiseptic remedies are the cause of these complications. He injected into the animals, when in perfect health, one tenth or twelfth part of the quantity of corrosive sublimate, carbolic acid, etc., which is sufficient to kill them. He then extirpated one kidney, and examined it microscopically, with the result that in all cases he found glomerulonephritis. There was exudation between the glomerulus and the capsule, and the epithelium of the tubuli contorti was almost entirely destroyed. He found also fatty degeneration of the liver, the spleen, the heart-muscle, etc. The various antiseptic agents were found to be injurious in different degrees, corrosive sublimate being the most dangerous, then the others in the following order: iodoform, carbolic acid, salicylic acid, boric acid. Senger therefore recommends surgeons to avoid antiseptics in operations on the thorax and abdomen, and urges them either to employ sterilized water after the manner of your compatriot Mr. Lawson Tait, or a solution of salt. By bacteriological and pathological researches he proved, first, that this kills the streptococcus pyogenes aureus in twenty-eight minutes, and that the effect is independent of the degree of concentration, for a 5 per cent. solution is just as effectual as a 20 per cent. Secondly, he claims to have shown that chlorate of sodium does not in any way injure the organs, and that no dose is strong enough to kill any animal.

